# Presence of Inhibitory Glycinergic Transmission in Medium Spiny Neurons in the Nucleus Accumbens

**DOI:** 10.3389/fnmol.2018.00228

**Published:** 2018-07-11

**Authors:** Braulio Muñoz, Gonzalo E. Yevenes, Benjamin Förstera, David M. Lovinger, Luis G. Aguayo

**Affiliations:** ^1^Laboratory of Neurophysiology, Department of Physiology, Universidad de Concepción, Concepción, Chile; ^2^Laboratory of Neuropharmacology, Department of Physiology, Universidad de Concepción, Concepción, Chile; ^3^Laboratory for Integrative Neuroscience, National Institute on Alcohol Abuse and Alcoholism, National Institutes of Health, Bethesda, MD, United States

**Keywords:** mouse models, nucleus accumbens, synaptic transmission, glycine receptor, medium spiny neurons, propofol, ethanol

## Abstract

It is believed that the rewarding actions of drugs are mediated by dysregulation of the mesolimbic dopaminergic system leading to increased levels of dopamine in the nucleus accumbens (nAc). It is widely recognized that GABAergic transmission is critical for neuronal inhibition within nAc. However, it is currently unknown if medium spiny neurons (MSNs) also receive inhibition by means of glycinergic synaptic inputs. We used a combination of proteomic and electrophysiology studies to characterize the presence of glycinergic input into MSNs from nAc demonstrating the presence of glycine transmission into nAc. In D1 MSNs, we found low frequency glycinergic miniature inhibitory postsynaptic currents (mIPSCs) which were blocked by 1 μM strychnine (STN), insensitive to low (10, 50 mM) and high (100 mM) ethanol (EtOH) concentrations, but sensitive to 30 μM propofol. Optogenetic experiments confirmed the existence of STN-sensitive glycinergic IPSCs and suggest a contribution of GABA and glycine neurotransmitters to the IPSCs in nAc. The study reveals the presence of glycinergic transmission in a non-spinal region and opens the possibility of a novel mechanism for the regulation of the reward pathway.

## Introduction

Glycine receptors (GlyRs) are mainly found in the spinal cord and brain stem (Aguayo et al., 1996, 2004, 2014; Tapia and Aguayo, 1998; Eggers et al., 2000; Sebe et al., 2003; Bradaïa et al., 2004; Eggers and Berger, 2004; van Zundert et al., 2005; Mariqueo et al., 2014).

Previous studies with GlyR mutant mice strains (spastic, oscillator and spasmodic) having mutations in the GlyR α1 subunit (spasmodic and oscillator) or β subunit (spastic) demonstrated the inhibitory role of glycinergic phasic currents in sensorial processing (Buckwalter et al., 1994; Mülhardt et al., 1994; Ryan et al., 1994). In addition, these mice not only show an increased muscular tone, but also show a strong hyperekplexic phenotype with altered motor neuronal transmission due to an impairment of the glycinergic inhibitory mechanism, similar to some epileptogenic human diseases (Koch et al., 1996). For example, mutation of the gene that codes for the α1 subunit has been related to patients that exhibit hyperekplexia/seizure disease (Rees et al., 2001). Therefore, the dysregulation of glycinergic transmission can lead to several neurological pathologies.

On the other hand, potentiation of α1 GlyR by drugs of abuse, such as ethanol (EtOH) and propofol, may be relevant to human health since they can also produce motor, respiratory and cardiovascular alterations (Schmid et al., 1991; Ren and Greer, 2006; Chang and Martin, 2011; Krowicki and Kapusta, 2011; Moraga-Cid et al., 2011) by altering chloride permeability (Sebe et al., 2003; Eggers and Berger, 2004; Mariqueo et al., 2014; Wakita et al., 2016). Overall, the modulation of glycinergic transmission in spinal and brain stem neurons can induce sedative EtOH- and propofol-mediated behavior (Nguyen et al., 2009). For instance, α1 GlyRs are sensitive to low EtOH concentrations (30 mM) in brain stem neurons (Eggers et al., 2000; Sebe et al., 2003) and to propofol in spinal neurons (Wakita et al., 2016).

Several studies have reported the presence of synaptic and non-synaptic GlyRs in supra spinal regions, such as cerebellar nuclei (Husson et al., 2014), orbital frontal cortex (OFC) (Badanich et al., 2013), dorsal raphe nuclei (Maguire et al., 2014), medial prefrontal cortex (mPFC) (Lu and Ye, 2011; Salling and Harrison, 2014), ventral tegmental area (VTA) (Ye et al., 2001; Li et al., 2012) and nucleus accumbens (nAc) (Molander and Söderpalm, 2005; Chau et al., 2010; Jonsson et al., 2014; Förstera et al., 2017). Some of these GlyRs are sensitive to EtOH opening the possibility that these upper GlyRs might be relevant targets for EtOH brain actions (Ye et al., 2001; Badanich et al., 2013; Maguire et al., 2014). Indeed, we recently reported a new role of non-synaptic GlyRs modulating the EtOH inhibitory effects by chloride tonic currents specifically in D1 MSNs (Förstera et al., 2017).

In the present study, we use a combination of mouse brain slice electrophysiology and optogenetic techniques to examine the presence of glycinergic input to D1 MSNs in nAc. The data indicate the presence of functional synaptic GlyRs in this mesolimbic area. Furthermore, we found that these synaptic glycinergic currents were insensitive to low and high concentrations of EtOH, but potentiated by propofol.

## Materials and Methods

### Mice

Animal care and experimental protocols for this study were approved by the Institutional Animal Care and Use Committees at the Universidad de Concepción and followed the guidelines for ethical protocols and care of experimental animals established by NIH (National Institutes of Health, MD, USA). C57BL/6J mice are available from the Jackson Laboratory stock (Bar Harbor, ME, USA). GlyT2-eGFP (Zeilhofer et al., 2005), vGAT::ChR2-eYFP BAC transgenic mice (Zhao et al., 2011) and D1-GFP (Tg(Drd1a-EGFP)x60Gsat/Mmmh) transgenic mice were maintained in a C57BL/6J background. Mice were individually housed in groups of 2–4 mice on a 12-h light/dark cycle and given food and water *ad libitum*.

### Preparation of Brain Slices

C57BL/6J, vGAT::ChR2-eYFP and D1-GFP mice (PND 21–40) were decapitated as described earlier (Jun et al., 2011). The brain was quickly excised, placed in cutting solution containing (in mM): sucrose 194, NaCl 30, KCl 4.5, MgCl_2_ 1, NaHCO_3_ 26, NaH_2_PO_4_ 1.2, Glucose 10 (pH 7.4) saturated with 95% O_2_ and 5% CO_2_, glued to the chilled stage of a vibratome (Leica VT1200S, Germany), and sliced to a thickness of 300–400 μm. Slices were transferred to the aCSF solution containing (in mM): NaCl 124, KCl 4.5, MgCl_2_ 1, NaHCO_3_ 26, NaH_2_PO_4_ 1.2, Glucose 10, CaCl_2_ 2 (pH 7.4 and 310–320 mOsm) saturated with O_2_ at 30°C for 1 h. Then, the slices were transferred to the recording chamber with aCSF solution saturated with 95% O_2_ and 5% CO_2_ at RT. The slices were observed in a DIC-IR microscope using 10× and 40× objectives (Nikon Eclipse FN1, Japan).

### Electrophysiology

Coronal brain slices (300–400 μm) containing the nAc region were prepared from adult C57BL/6J, vGAT::ChR2-eYFP and D1-GFP mice (PND 21–30) as described earlier (Jun et al., 2011) and perfused (2 ml/min) with oxygenated (95% O_2_/5%CO_2_, RT) aCSF at 30–32°C. Whole-cell current recordings of accumbal neurons were performed using the voltage-clamp technique. Patch pipettes were prepared from filament-containing borosilicate micropipettes (World Precision Instruments) using a P-1000 micropipette puller (Sutter Instruments, Novato, CA, USA) having a 4 MΩ resistance used for whole cell recording. Series resistance was 80% compensated with the amplifier and only cells with a stable series resistance (about 12 MΩ and that did not change more than 15% during recording) were included for data analysis.

To isolate the glycinergic spontaneous and miniature synaptic activity, we used two internal solutions containing (mM, high Cl^−^): 120 KCl, 4.0 MgCl_2_, 10 HEPES, 10 BAPTA, 0.5 Na_2_-GTP and 2.0 Na_2_-ATP pH 7.4, 290–310 mOsmol, equilibrium potential ≈0 mV to record inward Cl^−^ current at the holding potential of −60 mV and (mM low Cl^−^): 120 KGluc, 10 KCl, 10 HEPES, 4.0 MgCl_2_, 10 BAPTA, 4.0 NaATP and 0.3 NaGTP adjusted to 300 mOsm pH = 7.4 equilibrium potential ≈−48 mV to record outward Cl^−^ current at more positive potentials and an aCSF solution saturated with O_2_/CO_2_. Glycinergic miniature inhibitory postsynaptic currents (mIPSCs) were pharmacologically isolated via bath application of the AMPA receptor antagonist; 6-Cyano-7-nitroquinoxaline-2,3-dione (CNQX, 10 μM), NMDA receptor antagonist; D-2-amino-5-phosphonovalerate (D-APV, 50 μM), the GABA_A_ antagonist; bicuculline (10 μM), and tetrodotoxin (TTX; 500 nM). Recordings were done using an Axopatch 200B amplifier (Axon Instruments, Union City, CA, USA) at a holding potential of −60 mV (KCl solution) or 20 mV (K-Gluc solution). Currents were displayed and stored on a personal computer using a 1322A Digidata (Axon Instruments, Union City, CA, USA), analyzed with Clampfit 10.1 (Axon Instruments, Union City, CA, USA) and MiniAnalysis 6.0 (Synaptosoft Inc.). Analysis of frequency (Hz), decay constant (ms), rise constant (ms) and amplitude (pA) were used to determine the effects of EtOH (10, 50 and 100 mM) and propofol (30 μM) on glycine mIPSCs. The decay constant of mIPSCs was fitted as single exponential and both rise and decay-phases were fitted between 10% and 90% of the maximal amplitude.

#### Electrically Evoked Synaptic Current

A cesium chloride internal pipette solution containing (in mM) 120 CsCl, 4.0 MgCl_2_, 10 HEPES, 10 BAPTA, 0.5 Na_2_-GTP and 2.0 Na_2_-ATP was used to record synaptic glycine mediated events. A concentric bipolar stimulating microelectrode (World Precision Instruments, Sarasota, FL, USA) was placed in the nAc adjacent to and in close proximity to the recording site. Stimulus pulses of 0.5 ms of duration were delivered to elicit a stable and submaximal evoked current with an isolated stimulator. For isolated evoked glycine IPSCs (eIPSC), bicuculline (10 μM), D-APV (50 μM) and CNQX (10 μM), were added to the aCSF and perfused via bath application. Glycine eIPSCs were measured at a holding potential of −60 mV. Decay constant (ms), amplitude (pA) and rise time (ms) of synaptic currents were measured to determine the effects of EtOH (100 mM) and propofol (30 μM) on evoked glycine IPSCs.

#### Optogenetics

Whole-cell patch-clamp recordings were made at 30–32°C at a holding potential of −60 mV using an Axopatch 200B amplifier (Axon Instruments, Union City, CA, USA). Patch pipettes (3–4 MΩ) were filled with internal solution containing: (in mM) 120 CsCl, 10 BAPTA, 4.0 MgCl_2_, 0.5 GTP, 2 ATP, and 10 HEPES (pH 7.40 adjusted with CsOH). QX-314 (5 mM) was added to block voltage-activated Na^+^ before establishing whole-cell recording, and the cell was allowed to stabilize for 2–5 min. Light-evoked inhibitory post-synaptic currents (oIPSCs) were elicited by a 500 μm optic fiber blue light (473 nm) illumination (1 ms duration) every 30 s. Glycinergic or GABAergic oIPSCs were isolated using bicuculline (10 μM) or strychnine (STN; 1 μM), respectively.

### Immunocytochemistry

**Figure 1:**
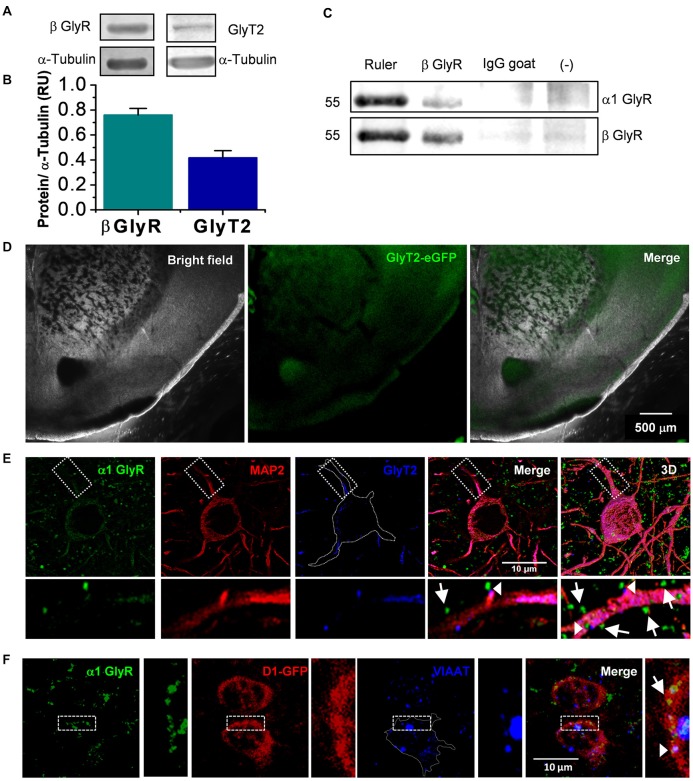
Presence of glycinergic proteins in nucleus accumbens (nAc). **(A)** Western blot from nAc of C57BL/6J mice for β Glycine receptor (GlyR) and GlyT2. **(B)** The graph summarizes normalized levels of GlyRβ subunit (*n* = 3) and GlyT2 (*n* = 6) in nAc. Bars are mean ± SEM. **(C)** Co-immunoprecipitation of β with α1 subunit reveals the presence of α1β heteromeric GlyR in the nAc. **(D)** Coronal brain slice from GlyT2-eGFP mice, which shows the presence of GlyT2 fibers in nAc. **(E)** Confocal microphotograph from coronal brain slice showing immunoreactivity to α1 GlyR (green), MAP2 (red) and GlyT2 (blue) in the nAc. Apposition of α1 GlyR with GlyT2 represents a synaptic receptor (arrowhead), while α1 GlyR alone are non-synaptic (arrow). **(F)** Confocal microphotograph from coronal brain slice from D1-GFP mice showing immunoreactivity to α1 GlyR (green), GFP (red) and VIAAT (blue) in the nAc. The colocalization of α1 GlyR with VIAAT represents a synaptic receptor (arrowhead), while α1 GlyR alone are non-synaptic (arrow). The scale bar represents 10 μm.

Brain slices were obtained as described above. Coronal slices (150–200 μm) containing nAc were fixed for 50 min with 4% PFA (Bioworld, USA). After three washes with 1× PBS, neurons were blocked and permeabilized with normal horse serum (10%) and Triton X-100 0.3% (Sigma) for 40 min with stirring. Slices were incubated with constant rocking (overnight) and a combination of primary antibodies: α1 GlyR (1:100, mouse monoclonal IgG, mAb4a clone; Cat. No. 146011BT, Synaptic System), MAP-2 (1:200, rabbit polyclonal IgG, H-300 clone, Cat. No. sc-20172, Santa Cruz biotechnology), and GlyT2 (1:200, goat polyclonal IgG, N-20 clone, sc-16704, Santa Cruz biotechnology). The specificity of mAb4a was confirmed in HEK cells transfected with α1, α2, α3 and β subunits. Additionally, we confirmed this in immature and mature spinal neurons expressing α1 only in the latter (Mariqueo et al., 2014). Cells were washed with 1× PBS and incubated (2 h) with a secondary anti-mouse, anti-goat and anti-rabbit antibody (Streptavidin Oregon Green; ExMax/EmMax = 496/524 nm, Cy3; ExMax/EmMax = 545/570 nm, Cy5; ExMax/EmMax = 649/670 nm, Molecular probes and Jackson Immuno Research, respectively) diluted 1:200 for 2 h with constant rocking. After five washes with 1× PBS, the preparations were mounted with Dako (DakoCytomation, USA) mounting solution. For VIAAT staining, the whole brain of a 1-month old D1-GFP mouse was fixed over night with Carnoy and mounted in paraffin to obtain 10 μm sections of the nAc. Primary antibodies for GFP (1:50, chicken polyclonal IgY, Cat. No. AB13970, Abcam) and VIAAT (1:100, guinea pig polyclonal antiserum; Cat. No. 131004, Synaptic System) were incubated overnight and combined with secondary antibodies (Alexa 633-anti-chicken and Cy3-anti-guinea pig 1:200, Jackson Labs) incubated for 3 h and mounted in Dako mounting solution containing DAPI (DakoCytomation, USA). Confocal images (1024 × 1024 pixels, pixel size was 313 nm) of a single optical section were acquired with 40×/1.3 n.a objective in a LSM700 laser scanning microscope and ZEN software suit (Zeiss, Oberkochen, Germany) in the CMA core facility at the Universidad de Concepción. Accumbal neurons in coronal slices were chosen randomly from view-fields presenting multiple cells exhibiting different levels of fluorescence. A 3D rendered image was generated from a z-stack of 16 optical sections (7.5 μm total optical thickness) for Figure 1E. Triple color immunofluorescent images were captured, processed, deconvoluted, rendered, stored and analyzed using the ZEN (Zeiss) ImageJ program (NIH).

### Western Blots

Tissue homogenates (100 μg; nAc) after detergent treatment (10 mM Tris-HCl pH 7.4, 0.25 M Sucrose, 10 mM NEM, Protease inhibitor cocktail 1×) were subjected to electrophoresis on 10% SDS–PAGE gels. Proteins were blotted onto nitrocellulose membranes (Biorad) and blocked with 5% milk in 1× TBS, 0.1% Tween 20 for 1 h with constant rocking. Subsequently, the membranes were incubated with primary β GlyR (1:1000, mouse monoclonal IgG, 299E7 clone, Cat. No. 146211, Synaptic System), GlyT2 (1:200, goat polyclonal IgG, N-20 clone, sc-16704, Santa Cruz biotechnology), α1 GlyR (1:1000, mouse polyclonal IgG, mAb2b clone, Cat. No. 146111, Synaptic System) and anti α-tubulin (1:3000, mouse monoclonal IgG, B-5–1–2 clone, Cat. No. T5168, Sigma) antibodies for 1–2 h. After washes with 1× TBS and 0.1% Tween 20, membranes were incubated for 1 h with anti-mouse and goat secondary antibodies conjugated to HRP (1:5000, Santa Cruz). The immunoreactivity of the proteins was detected and visualized with ECL Plus Western Blotting Detection System (PerkinElmer, MA, USA). Levels of α-tubulin were used as a loading control. The Western blot was quantified by using the “ImageJ” (NIH) program.

### Co-immunoprecipitation

For co-immunoprecipitation experiments, nAc homogenates (200 μg) were prewashed after lysis buffer treatment (10 mM Tris-HCl pH 7.4, 0.25 M Sucrose, 10 mM NEM, Protease inhibitor cocktail 1×) using 40 μl of Protein A/G plus Agarose (Santa Cruz Biotechnology) and 500 μl lysis buffer without protease inhibitors, incubated with constant agitation for 2 h at 4°C and centrifuged at 2000 *g* for 5 min. The resulting supernatant was the prewashed lysate. The lysate was incubated with anti-GlyR β antibody (1 μg, mouse monoclonal IgG, 299E7 clone, Cat. No. 146211, Synaptic System) and normal goat IgG antibody (400 ng, sc-2028, Santa cruz Biotechnology) with constant rocking at 4°C for at least 1.5 h. Then the equilibrate resin (40 μl) was added to the lysates, incubated with constant agitation for 2 h at 4°C and then centrifuged at 2000 *g* for 5 min. The resulting pellet was washed three times and the co-immunoprecipitated material was recovered and heated at 95°C for 10 min and prepared to perform a Western blot.

### Reagents

Bicuculline, STN and propofol were obtained from Sigma-Aldrich (USA). D-APV and CNQX were purchased from Tocris (Bristol, UK). TTX was purchased from Alomone labs (Jerusalem, Israel). Ethanol was purchased from Merck Millipore (USA).

### Sample Size

The target number of samples in each group for biochemistry and electrophysiological experiments was determined based on numbers reported in published studies (Aguayo et al., 2014; Mariqueo et al., 2014; Förstera et al., 2017).

### Replication

All sample sizes indicated in figures for electrophysiological experiments represent biological replicates. The biochemistry experiments (western blot and immunocytochemistry) were repeated at least three times.

### Data Analyses

Unless otherwise indicated, data were presented as mean ± SEM. The analyses were performed using two-tailed unpaired, two-tailed paired Student’s *t*-tests following an F-test to confirm similar variances for all the data. Non-normally distributed data were analyzed using two-tailed Mann-Whitney signed rank tests. Statistical analyses were performed with Origin 6.0 and 8.0 (Microcal Inc. Northampton, MA, USA). Alpha was always set at *p* < 0.05. Values for *p < 0.05 was considered statistically significant.

## Results

### The Presence of Several Synaptic Proteins in nAc Supports the Existence of Glycinergic Transmission

The results obtained with the western blot experiments support the presence of the β subunit necessary for anchoring α GlyRs to the postsynaptic site (Grudzinska et al., 2005) and GlyT2, a presynaptic glycine reuptake transporter (Bradaïa et al., 2004), in the nAc (Figures 1A,B). Also, co-immunoprecipitation data support the presence of α1β GlyR complexes in the same region (Figure 1C). Furthermore, coronal slices obtained from GlyT2-eGFP mice indicated the presence of green fluorescence associated to synaptic glycine transporters in the nAc (Figure 1D), similar to studies in the dorsal basal ganglia (Zeilhofer et al., 2005). In addition, immunocytochemistry in nAc slices showed that some α1 GlyR (green) co-localized with GlyT2 (blue) supporting presence of synaptic α1 GlyR (arrow heads, Figure 1E). Also, the apposition between vesicular inhibitory amino acid transporter (VIAAT) (blue) and α1 GlyR (green) further confirmed the presence of synaptic GlyRs (arrow head, Figure 1F).

### Presence of Glycine-Mediated IPSCs in Nucleus Accumbens

**Figure 2:**
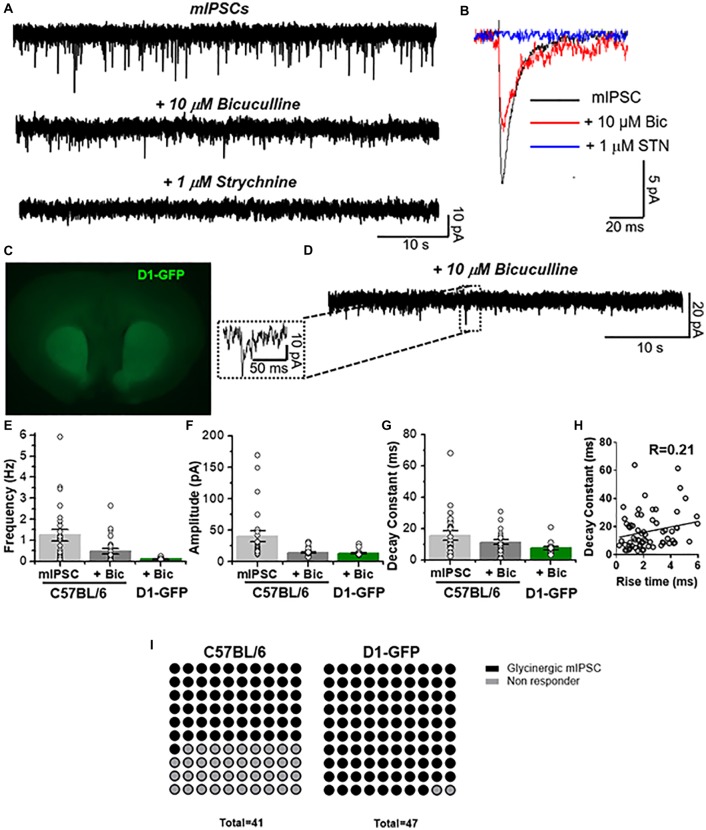
Presence of glycinergic transmission in medium spiny neurons (MSNs). **(A)** Representative synaptic current traces from C57BL/6J mice showing the total miniature inhibitory postsynaptic currents (mIPSC) and the pharmacologically isolated glycinergic mIPSC (recorded in presence of TTX (500 nM), bicuculline (10 μM), CNQX (10 μM) and D-APV (50 μM)) and glycinergic mIPSC blocked by 1 μM strychnine (STN). **(B)** Average traces of mIPSC (black line), glycinergic mIPSC (red, denominated + bicuculline) and STN blocked (blue). **(C)** Coronal brain slice from D1-GFP mice showing the fluorescence in dorsal striatum and nucleus accumbens. **(D)** Representative synaptic traces from a D1 MSN showing glycinergic mIPSCs recorded in presence of TTX (500 nM), bicuculline (10 μM), CNQX (10 μM) and D-APV (50 μM). **(E–G)** The graphs summarize synaptic event properties of frequency **(E)**, amplitude **(F)** and decay constant **(G)** from mIPSCs and glycinergic synaptic current in accumbal neurons from C57 and D1-GFP mice. **(H)** The graph shows the relation between rise time constant (10%–90%) vs. decay time constant (10%–90%) of glycinergic synaptic events obtained from 4 D1 MSNs. No correlation was found (*R* = 0.21, Spearman’s correlation rank test: *p* = 0.0727). Data are mean ± SEM (*n* = 25 C57, *n* = 15 D1-GFP). **(I)** Dot plots summarize the MSNs that have glycinergic synaptic currents and those where no currents were present. GlyR-mediated synaptic currents were found in 61% of MSNs from C57BL/6 mice and in 98% of MSNs from D1-GFP mice.

The previous data showed the presence of several biochemical and structural components that might support functional glycinergic neurotransmission in accumbal neurons. Next, we performed patch clamp recordings in nAc slices from C57BL/6J mice and found the presence of fast-decaying, low amplitude and frequency synaptic currents in 25 of 41 registered accumbal neurons that were blocked by a low concentration of STN, corresponding to glycinergic neurotransmission (Figures 2A,B,I). Throughout the manuscript we labeled glycinergic mIPSCs as “+10 μM bicuculline” because these events were recorded under a cocktail containing a GABA_A_R antagonist (see “Materials and Methods” section). These synaptic currents were still found in the presence of 10 μM mecamylamine, a nicotinic receptor antagonist (data not shown), negating the possibility that these responses were due to activation of these excitatory, cationic carrying receptors. To characterize the type of accumbal neurons that receive the glycinergic input, we used D1-GFP mice (Figure 2C). In GFP positive MSNs we also detected glycine-mediated mIPSCs with an event frequency of 0.11 ± 0.01 Hz (*n* = 15, Figure 2E) in 46 of 47 D1 MSNs (Figure 2I). The amplitude of the unitary current was 13 ± 1 pA while the decay displayed a time constant of 7.5 ± 1 ms (Figures 2F,G). Furthermore, the data did not show any correlation (*R* = 0.21, *p* = 0.0727, Spearman’s correlation rank test) between decay and rise constant for glycinergic mIPSC (Figure 2H), supporting earlier reports that these types of events are synaptic in nature and that the properties are not altered by membrane filtering (van Zundert et al., 2004).

**Figure 3:**
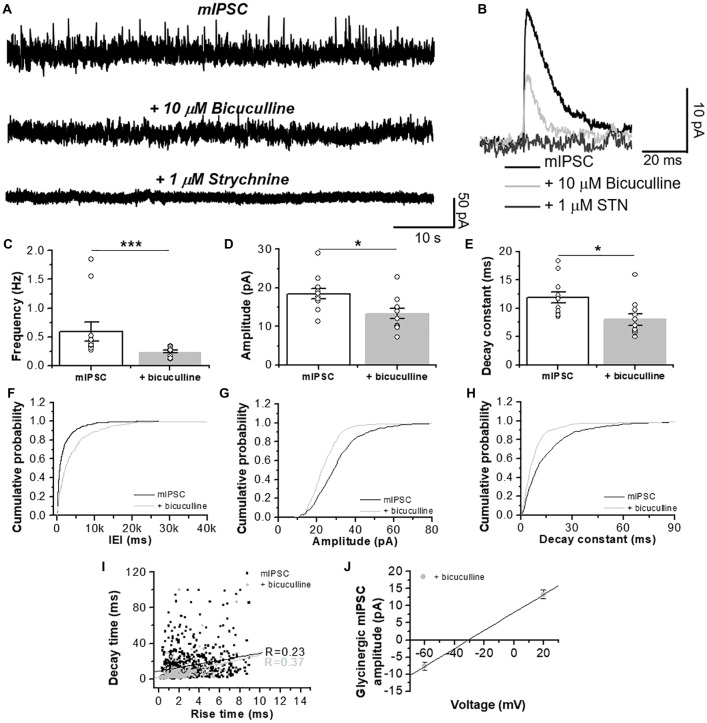
GlyR-mediated chloride currents in D1 MSNs are inhibited by STN. **(A)** Representative synaptic traces in presence of CNQX (10 μM) and D-APV (50 μM), plus bicuculline (10 μM) and STN (1 μM) recorded at a +20 mV holding potential in neurons from D1-GFP mice. **(B)** Average traces of total pharmacologically isolated mIPSC (black line), mIPSC plus 10 μM bicuculline (light gray) and plus 1 μM STN (dark gray). **(C–E)** Graphs summarize the chloride current properties in D1 MSNs showing a decrease in frequency (*p* = 0.001, *t*_20_ = 2.109, Unpaired Student’s *t*-test, Mann-Whitney test), amplitude (*p* = 0.013, *t*_20_ = 2.711 Unpaired Student’s *t*-test) and decay constant (*p* = 0.011, *t*_20_ = 2.8 Unpaired Student’s *t*-test). Those currents were sensitive to 1 μM STN. **(F–H)** The graphs are cumulative histograms of frequency, amplitude and decay time (10%–90%) in D1 MSNs. **(I)** The graph shows lack of relationship between rise time constant (10%–90%) vs. decay time constant (10%–90%) of chloride synaptic events for GABA (*R* = 0.23) and glycine mIPSCs (*R* = 0.37). **(J)** The graph shows the relationship between voltage holding (−60 and +20 mV) and the amplitude of the GlyR-mediated mIPSCs. Using the chord conductance equation we calculated that the reversal potential of glycinergic mIPSC was −31 mV. The data are mean ± SEM. (*n* = 11). ns *p* < 0.05, *p < 0.05, ***p < 0.001.

To further characterize the ionic nature of these synaptic currents, we used a low internal chloride concentration to elicit an outward current at positive potentials (i.e., +20 mV). Because a potential cationic contribution possibly produced by a cholinergic component is minimal at this holding potential (Na^+^ reversal potential is approximately 0 mV, see “Materials and Methods” section), the synaptic response observed should be primarily carried by Cl^−^ ions. The data in Figure 3 shows the presence of total mIPSCs for GABA_A_- and GlyR-mediated currents in a D1 MSN (Figures 3A,B). Application of bicuculline (10 μM) caused a reduction in the frequency, amplitude and decay time constant indicating that the glycinergic component is a smaller fraction of the total inhibitory synaptic current (Figures 3C–H). The events identified as glycinergic, recorded in the presence of bicuculline, were completely inhibited by STN. Furthermore, the current noise detected at the level of the holding current was reduced by STN (Figure 3A) suggesting the presence of a GlyR-mediated tonic current; results that are in agreement with those recently reported (Förstera et al., 2017). Similar to the data in Figure 2H, no correlation was found between decay time and rise constant (Figure 3I). Interestingly, we found a linear relationship between voltage holding and mIPSC amplitude, with an estimated reversal potential at approximately −30 mV (Figure 3J), which is close to the predicted reversal potential for Cl^−^.

### Optogenetic Activation of Accumbal GABAergic Interneurons Elicits Mixed Inhibitory Synaptic Responses

**Figure 4:**
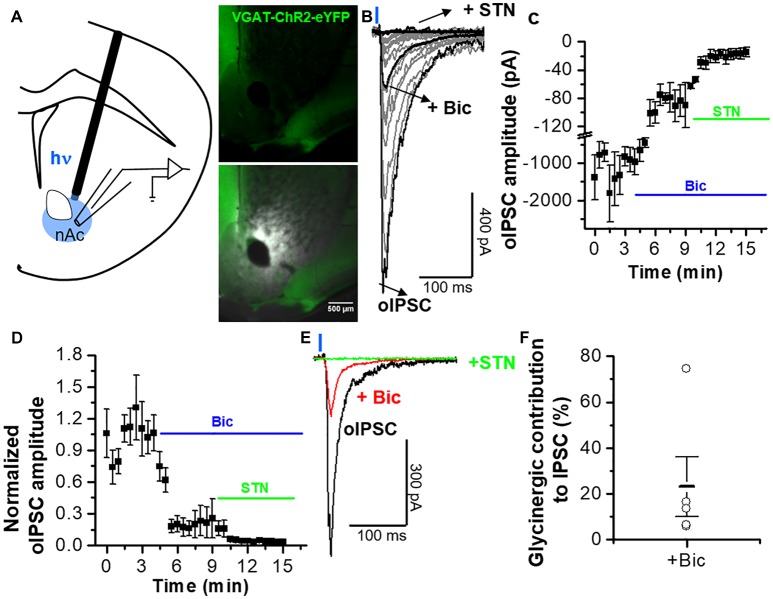
Optogenetically activated inhibitory synaptic transmission in MSNs from nAc. **(A)** Schematic illustrating oIPSCs recording evoked by 1-ms, 473-nm light focal stimulation. Distribution of vGAT-ChR2-eYFP expression observed in coronal brain slice. **(B)** Average oIPSC traces per cell (gray) of five neurons from vGAT-ChR2-eYFP mice. Black traces represent the average oIPSC for total, plus 10 μM bicuculline, and plus bicuculline/STN. **(C)** Graph represents average of IPSC amplitudes during the application of bicuculline and STN. **(D)** Time course of the oIPSCs with a combination of bicuculline (10 μM) and STN (1 μM) applied at 4 and 9 min, respectively, after the recording started. **(E)** Average traces of control conditions (black), during application of bicuculline (red), and in the combined presence of STN and bicuculline (green) showing the difference in the amplitude and decay kinetics of the GABAergic and glycinergic IPSC components. **(F)** Open circles represent the glycinergic IPSC component in individual cells, indicating an average contribution of GlyR-mediated inhibitory current of 23 ± 13% to the oIPSC. The data are mean ± SEM, *n* = 5.

The previous results indicate the presence of GABA and glycinergic inputs into nAc D1 MSNs, possibly mediated by the release of GABA and glycine, as previously suggested to occur in other brain regions (Dugué et al., 2005; Husson et al., 2014). To evaluate the existence of a similar activity in nAc, we used the whole-cell voltage-clamp configuration to record MSNs in brain slices from vGAT-ChR2-eYFP mice stimulated with 1 ms illumination (the focal region near the recording area, Figure 4A). The optogenetic stimulations lead to the generation of inhibitory synaptic currents (oIPSC total) (Figure 4B) with an amplitude of −1323 ± 523 pA, *n* = 6) at 2.5 min of recording (Figure 4C). Bath application of 10 μM bicuculline decreased the amplitude of the total oIPSC up to a steady state level (Figure 4B). The amplitude of the isolated glycinergic oIPSC (oIPSC) was reduced to −91 ± 22 pA at 8 min of recording in the presence of bicuculline (Figure 4C). Finally, co-application of bicuculline and 1 μM STN blocked all the light-evoked synaptic current (−15 ± 7 pA at 15 min; Figures 4B,C). Additional normalized data in presence of bicuculline and STN is shown as a relation to GABAergic amplitude and glycinergic oIPSCs (oIPSC total; Figure 4D). In fact, the inhibition of GABAergic oIPSCs by bicuculline reduced the amplitude of the current to 23 ± 14%, which should be the contribution of glycinergic oIPSCs (Figures 4D,F), with individual variable contributions from cell to cell between 74%–6% (Figure 4F). The light-evoked synaptic current in presence of bicuculline was blocked by STN (3 ± 1%; Figures 4D,E). These results provide the first evidence for functional inhibitory neurotransmission at interneuronal synapses establishing the presence of a glycinergic input to MSNs in nAc.

### Glycinergic Neurotransmission Was Sensitive to Propofol but Not EtOH

**Figure 5:**
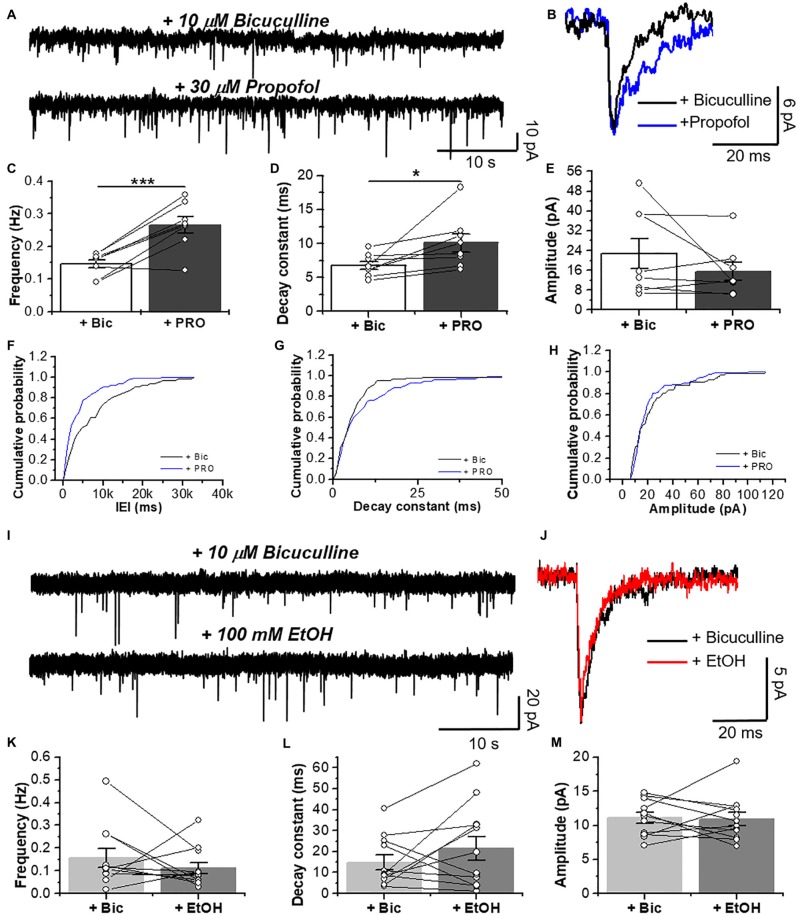
Glycinergic mIPSC in D1 MSNs are potentiated by propofol but not ethanol EtOH. **(A)** Representative synaptic current traces from D1 MSNs showing glycinergic mIPSCs recorded in the absence and presence of 30 μM propofol. **(B)** Average traces of glycinergic synaptic event in (black line), and plus 30 μM propofol (blue line). **(C–E)** Graphs summarize the glycinergic current properties in D1-GFP mice. Data show an increase in frequency (*p* = 0.0009, *t*_7_ = 5.506) and decay constant (*p* = 0.041, *t*_7_ = 2.49) of glycinergic mIPSCs by 30 μM propofol (*n* = 8). **(F–H)** The graphs are cumulative histograms for frequency **(F)**, decay time (10%–90%) **(G)** and amplitude **(H)** in D1 MSNs. **(I)** Representative synaptic current traces from D1-GFP mice showing glycinergic mIPSCs recorded in the absence and presence of 100 mM EtOH. **(J)** Average traces of GlyR-mediated synaptic event (black line) plus 100 mM EtOH (red line). **(K–M)** Graphs summarize the glycinergic current properties in D1 MSNs. Data shows no differences in the synaptic parameters with EtOH (*n*=12), ns *p* > 0.05, *p < 0.05, ***p < 0.001, paired-sample *t*-test.

After blocking AMPA-, NMDA- and GABA_A_-mediated neurotransmissions, we performed patch clamp recordings in D1 MSNs and we examined the effects of 30 μM propofol, a glycinergic modulator (Moraga-Cid et al., 2011), on spontaneous GlyR-mediated mIPSCs (Figure 5) and electrically-evoked glycine IPSCs (eIPSCs; Figure 7). For example, application of 30 μM propofol led to a significant increase in glycinergic frequency in D1 MSNs (0.15 ± 0.01 Hz vs. 0.27 ± 0.03 Hz, ***p < 0.001, paired-sample *t*-test, *n* = 8; Figures 5A,C,F). The decay constant was also increased (6.6 ± 0.6 ms vs. 10.3 ± 1.5 ms, *p < 0.05, paired-sample *t*-test, *n* = 8; Figures 5B,D,G, but no changes were observed in the amplitude (Figures 5B,E,H).

**Figure 6:**
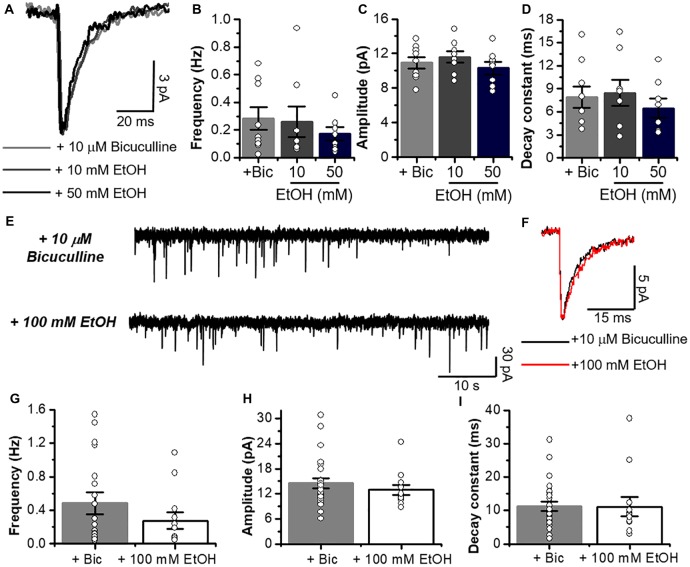
Glycinergic mIPSCs properties are not altered by low and high concentrations of EtOH. **(A)** Average synaptic event traces of glycinergic mIPSC from C57BL/6J MSNs in presence of a cocktail containing 10 μM bicuculline (gray line), plus 10 mM (dark gray line) and 50 mM EtOH (black line). **(B–D)** Graphs summarize the glycinergic current properties in MSNs from C57 mice (Bicuculline, *n* = 9; 10 and 50 mM, *n* = 8). Data show that low and high concentrations of EtOH do not affect frequency (Glycinergic mIPSC vs. 10 mM; *p* = 0.88, *t*_15_ = 0.1536 and Glycinergic mIPSC vs. 50 mM; *p* = 0.2819, *t*_15_ = 1.116), amplitude (Glycinergic mIPSC vs. 10 mM; *p* = 0.476, *t*_15_ = 0.731 and Glycinergic mIPSC vs. 50 mM; *p* = 0.5437, *t*_15_ = 0.6214) and decay constant (Glycinergic mIPSC vs. 10 mM; *p* = 0.8123, *t*_15_ = 0.2417 and Glycinergic mIPSC vs. 50 mM; *p* = 0.4534, *t*_15_ = 0.7698). **(E)** Representative synaptic current traces from C57BL/6J mice showing the glycinergic mIPSC recorded in the absence and presence of 100 mM EtOH. **(F)** Average traces of glycinergic mIPSC (black line) and plus 100 mM EtOH (red). **(G–I)** Graphs summarize the glycinergic current properties in C57BL/6J mice. Data show no differences in frequency (Glycinergic mIPSC vs. 100 mM; *p* = 0.31, *t*_35_ = 1.028), amplitude (Glycinergic mIPSC vs. 100 mM; *p* = 0.4116, *t*_35_ = 0.8311) and decay constant (Glycinergic mIPSC vs. 100 mM; *p* = 0.9539, *t*_35_ = 0.05822) with high EtOH concentration. *n* = 25 for control; *n* = 12 for EtOH. Unpaired Student’s *t*-test.

**Figure 7:**
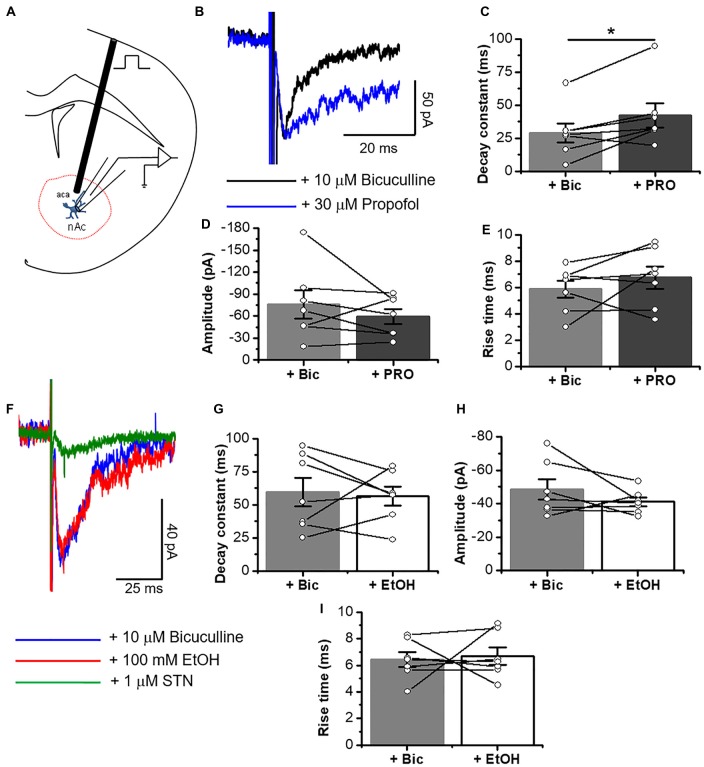
Electrically elicited eIPSCs are potentiated by propofol in D1 MSNs. **(A)** Schematic figure of coronal brain slice showing the recording of IPSCs evoked by focal electric stimulation (0.5 ms duration). **(B)** Representative electrically evoked synaptic traces in presence of an inhibitory cocktail for excitatory transmission containing 10 μM bicuculline (black line) alone and plus 30 μM propofol (blue line) in D1 MSNs. **(C–E)** Graphs summarize the effects of propofol on decay constant **(C)**, amplitude **(D)** and rise time **(E)** in D1 MSNs. The results show a significant increase in decay constant (*p* = 0.0316, *t*_6_ = 2.79), but without changes in amplitude (*p* = 0.3152, *t*_6_ = 1.096) and rise time (*p* = 0.3148, *t*_6_ = 1.97) of glycinergic eIPSC (*n* = 7). **(F)** Representative electrically evoked synaptic traces in presence of a cocktail with 10 μM bicuculline (blue line), 100 mM EtOH (red line) and 1 μM STN (green line) in neurons from D1-GFP mice. **(G–I)** Graphs show that 100 mM EtOH did not affect either decay constant (*p* = 0.7806, *t*_6_ = 0.2913), amplitude (*p* = 0.2418, *t*_6_ = 1.299) or rise time (*p* = 0.7939, *t*_6_ = 0.2732) in D1 MSNs (*n*=7). Data are mean ± SEM. *p < 0.05, paired-sample *t*-test.

On the other hand, EtOH did not affect glycinergic mIPSC parameters at any of the concentrations used (Figures 5I–M, 6A–I). Contrary to ethanol, application of propofol (30 μM, 7B) significantly increased the decay constant of glycinergic eIPSCs (29.1 ± 7.2 ms to 42.2 ± 9.2 ms, *p < 0.05, paired-sample *t*-test, *n* = 7), but had no effect on the amplitude and rise time (Figures 7B–F). On the other hand, the electrically evoked glycinergic current in D1 MSNs was not affected by 100 mM ethanol (Figures 7G–I).

**Figure 8:**
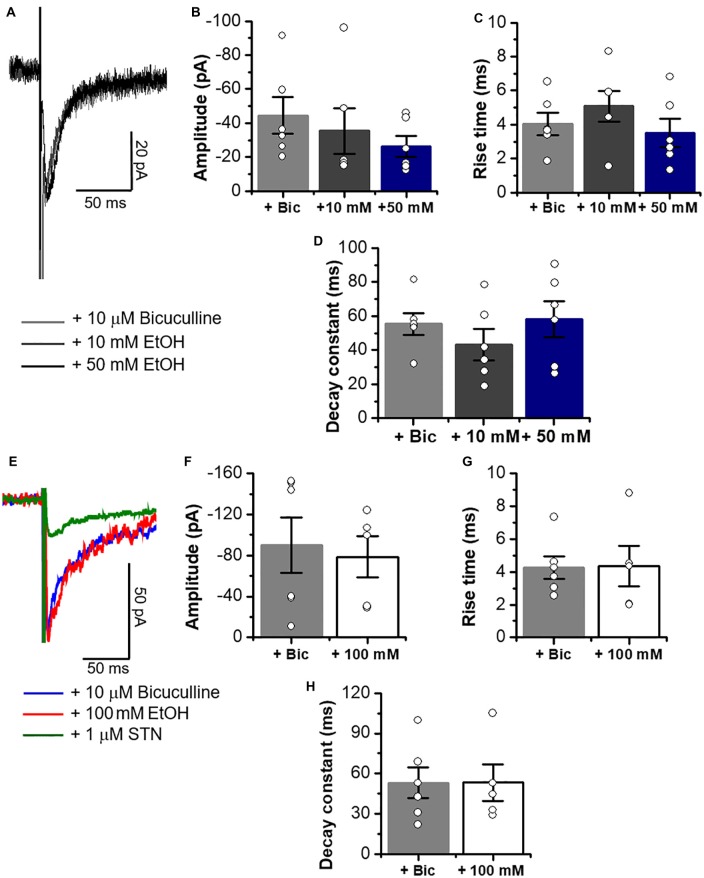
Glycinergic eIPSCs properties in C57BL/6J mice are not altered by 10, 50 and 100 mM EtOH. **(A)** Representative electrically evoked synaptic traces in presence of bicuculline (10 μM), CNQX (10 μM) and D-APV (50 μM) (eIPSCs, gray line), plus 10 mM (dark gray line) and 50 mM EtOH (black line) in neurons from C57BL/6J mice. **(B–D)** Graphs summarize the lack of EtOH effects on amplitude (Control vs. 10 mM; *p* = 0.6059, *t*_10_ = 0.5327; Control vs. 50 mM; *p* = 0.1766, *t*_10_ = 1.454), rise time (Control vs. 10 mM; *p* = 0.3785, *t*_10_ = 0.9215; Control vs. 50 mM; *p* = 0.6369, *t*_10_ = 0.4867) and decay constant (Control vs. 10 mM; *p* = 0.3004, *t*_10_ = 1.092; Control vs. 50 mM; *p* = 0.831, *t*_10_ = 0.2191) in MSNs (*n* = 6). **(E)** Representative electrically evoked synaptic traces in presence of a cocktail of inhibitors for excitatory transmission plus 10 μM bicuculline (blue line), plus 100 mM EtOH (red line) and 1 μM STN (green line) in MSNs from C57BL/6J mice. **(F–H)** Graphs show that a high concentration of EtOH does not affect the amplitude (*p* = 0.7529, *t*_9_ = 0.3246), rise time (*p* = 0.9499, *t*_9_ = 0.0646) or decay constant (*p* = 0.9952, *t*_9_ = 0.0062) of glycinergic eIPSCs in MSNs (*n* = 6 control, *n* = 5,100 mM EtOH). Unpaired Student’s *t*-test. Bars are mean ± SEM.

The data in Figure 8 show that the properties of electrically elicited glycinergic eIPSC in C57 mice were not affected by 10–100 mM EtOH as suggested by the presence of similar current properties before and during application (amplitude, rise time and decay constant) in MSNs (Figures 8A–H).

## Discussion

### Presence of Glycinergic Neurotransmission in the Mesolimbic System

The GABA_A_R mediated Cl^−^ current is considered to provide the main inhibitory neurotransmission in the brain and is a main molecular site for the action of several drugs acting in the mesolimbic dopamine system (Nestler, 2005). The present study provides evidence that supports the existence of an additional, although smaller inhibitory transmission component, mediated by GlyRs in the nAc, a critical region for brain reward. This conclusion is based on the presence of GlyT2, a presynaptic glycine transporter and the β GlyR subunit, which is well known to anchor, together with gephyrin, the GlyR to the postsynaptic region (Weltzien et al., 2012; Zeilhofer et al., 2005). Furthermore, co-immunoprecipitation of α1 together to β suggested the presence of the α1β GlyR complex, which was previously found to be mainly localized at synaptic sites (Grudzinska et al., 2005; Zeilhofer et al., 2018). In addition, using confocal microscopy we found that GlyT2 and VIAAT immunoreactivity apposed with α1 GlyR subunits in nAc neurons. The above results which support the presence of synaptic α1β GlyRs in the nAc is in good agreement with a previous histological study that showed the existence of some GlyT2 fibers present in this region (Zeilhofer et al., 2005). Additionally, our electrophysiological results show unambiguously the presence of synaptic currents in accumbal neurons having all the properties of glycinergic transmission: fast kinetics, mecamylamine resistance and blockade by low concentrations of STN (van Zundert et al., 2005; Aguayo et al., 2014; Mariqueo et al., 2014; Wakita et al., 2016). Also, these results suggest that GABAergic D1 and D2 MSNs in nAc receive glycinergic inputs from a still unknown origin. Overall, we found that approximately 60% of the MSNs we examined presented glycinergic IPSCs and this heterogeneity may be related to the distinct types of neurons present in nAc (Russo and Nestler, 2013). Indeed, D1 MSNs presented α1 synaptic GlyR apposed to VIAAT, which correlated with the ubiquitous presence of IPSC in D1 positive neurons (≈98%), supporting the notion that D1 MSNs regulate their inhibitory function by both GABA and glycine neurotransmissions (Figure 1).

A potential co-release of GABA and glycine in the nAc is not unexpected because it was reported to occur in several other brain regions (Jonas et al., 1998; Wojcik et al., 2006; Seddik et al., 2007; Lu et al., 2008; Husson et al., 2014). In the nAc, the phasic inhibition appears to be provided by GABA_A_R- and GlyR-mediated neurotransmission, with a glycinergic contribution of approximately 20% of the global inhibitory component. Therefore, our results reporting the presence of glycine-mediated IPSCs in nAc identifies a new region in addition to those reported in other critical brain regions, such as cerebellum and dorsal raphe nuclei (Husson et al., 2014; Maguire et al., 2014). Altogether, these findings support the notion of glycinergic inhibitory neurotransmission in the mesolimbic region.

### The Pharmacological Properties of Glycinergic IPSCs in the nAc

Previous reports have shown the sensitivity of glycinergic IPSCs to several ligands. In spinal neurons, for example, glycinergic neurotransmission is sensitive to STN, EtOH, zinc and general anesthetics (Aguayo et al., 2004, 2014; Smart et al., 2004; Mariqueo et al., 2014; Wakita et al., 2016). The GlyRs present in other brain regions seem to exhibit a similar pharmacology with their inhibition by low STN being their main signature (Husson et al., 2014; Maguire et al., 2014; Salling and Harrison, 2014). Our data confirm the sensitivity of accumbal IPSCs to 1 μM STN, which was enough to inhibit all the glycine-mediated IPSCs. Furthermore, the synaptic currents activated by optogenetic and electrical stimulations were sensitive to STN, similar to previous reports (Husson et al., 2014; Foster et al., 2015). Altogether, these observations support the existence of STN-sensitive synaptic currents in accumbal MSNs. Recording glycinergic currents in presence of mecamylamine and near the reversal potential for excitatory neurotransmissions further support this conclusion (Figure 3A).

Synaptic GlyRs in the nAc appear to be mainly composed of α1β heteropentameric conformations. To further characterize the likely composition of these GlyRs, we evaluated the sensitivity of isolated glycinergic IPSCs to two classic allosteric modulators: propofol (Moraga-Cid et al., 2011; Wakita et al., 2016) and EtOH (Aguayo et al., 2014; Mariqueo et al., 2014; Burgos et al., 2015a,b; Naito et al., 2015). We found that D1 MSNs express synaptic GlyRs that are sensitive to propofol. Indeed, propofol was also able to increase the frequency of mIPSC, likely suggesting a presynaptic action (Mariqueo et al., 2014; Wakita et al., 2016). Additionally, the significant increase in the decay constant of glycinergic IPSCs indicates a direct modulation of postsynaptic α1 GlyRs, which is related to the potentiation of glycine-mediated chloride currents (Moraga-Cid et al., 2011). Presently, not much is known about the addictive properties of propofol, but some reports have determined a relationship between the use of this anesthetic and the development of substance-abuse (Luck and Hedrick, 2004; Roussin et al., 2007; Klausz et al., 2009; Wilson et al., 2010).

On the other hand, the applications of low and high concentrations of EtOH did not change the synaptic properties of glycine-mediated IPSCs suggesting that EtOH actions on accumbal GlyRs are mediated by non-synaptic receptors that are indeed affected by EtOH (Maguire et al., 2014; Förstera et al., 2017). Furthermore, the effects of EtOH in the nAc can lead to a GlyR-dependent release of dopamine, a mechanism that could play a role in its addictive actions (Li et al., 2012; Jonsson et al., 2014; Blednov et al., 2015). In summary, glycinergic IPSCs in the nAc are sensitive to propofol, but resistant to the effects of EtOH.

### The Potential Functional Impact of Glycinergic Input to D1 MSNs

Inhibitory neurotransmission is essential in the regulation of neural circitry and the main inhibitory neurotransmitter in the mesolimbic dopamine system is GABA (Hyman et al., 2006). However, previous studies using pharmacological and intracerebral dialysis techniques have indicated that GlyRs in nAc and VTA are important for the release of dopamine and addiction-mediated behaviors (Molander et al., 2005; Li et al., 2012). This notion is in line with the widely recognized view that reward-related learning is associated with activation of the direct nAc-VTA pathway (Macpherson et al., 2014). Moreover, the activation of D1 MSNs appears to be related to a high preference for cocaine, whereas activation of D2 MSNs results in aversive behavior (Lenz and Lobo, 2013; Nakanishi et al., 2014).

The present data support the notion that glycinergic neurotransmission in the nAc contributes to the excitatory/inhibitory balance in this region. Specifically, the presence of a glycinergic input to D1 MSNs suggests that it may be involved in regulation of reward-related learning. Indeed, it was reported that D1 MSNs are important in the maintenance of propofol self-administration (Lian et al., 2013). Moreover, the systemic administration of a glucocorticoid receptor agonist in the nAc can regulate propofol self-administration behavior, altering the D1 receptor and c-Fos expression in rats (Wu et al., 2016, 2018). Also, propofol increases DeltaFosB in nAc mediated by D1 receptors (Xiong et al., 2011) thus linking the rewarding effect of propofol directly to D1 MSNs.

In conclusion, this study provides the first evidence of functional glycinergic neurotransmission input to D1 MSNs that is sensitive to propofol, suggesting that synaptic GlyR are involved in regulating the actions of excitatory-inhibitory balance as well as the effects of propofol and possibly other drugs of abuse. In addition, these findings suggest a new cellular target and a potentially effective pharmacotherapeutic point of attack for the prevention and treatment of propofol abuse. With regards to ethanol actions on the direct pathway, it would appear that its effect on tonic inhibition is the one related to addictive behavior because non-synaptic GlyRs in the nAc are modulated by ethanol (Förstera et al., 2017) whereas synaptic ones are not.

## Author Contributions

BM, BF, GY, DL and LGA designed experiments, discussed the results, contributed to all stages of manuscript preparation and editing. BM performed all the experiments. BF performed IHC for VIAAT. All authors revised and approved the final version of the manuscript.

## Conflict of Interest Statement

The authors declare that the research was conducted in the absence of any commercial or financial relationships that could be construed as a potential conflict of interest.
